# Acute compartment syndrome secondary to anterolateral thigh flap harvesting in a pediatric patient: A case report

**DOI:** 10.1097/MD.0000000000021216

**Published:** 2020-07-10

**Authors:** Wenrui Qu, Junbo Pan, Hongjuan Jin, Xuejie Wang, Heng Tian

**Affiliations:** a Department of Hand surgery, the Second Hospital of Jilin University, 218 Ziqiang St, Changchun, Jilin province; b Department of Hand and foot surgery, the affiliated Hospital of Yanzhou University, Yanzhou, Jiangsu province; c Department of Plastic and Reconstructive Surgery, the First Hospital of Jilin University, Changchun, China.

**Keywords:** anterolateral thigh flap, compartment syndrome, complications

## Abstract

**Introduction:**

The anterolateral thigh (ALT) flap is the most popularly used flap for major soft tissue reconstruction. Although it is widely used, acute compartment syndrome (ACS) in pediatric patients has rarely been reported in the literature. We herein reported a case of ACS in a 6-year-old girl after ALT flap harvest with direct closure of the donor site.

**Patient concerns:**

A 6-year-old girl was admitted to the Second Hospital of Jilin University with crush injury on the palmar aspect of the right hand and wrist.

**Diagnosis:**

Examination showed soft tissue defect of the hand and wrist, damage on the thenar muscles, lightly crushed flexor tendons, crushed median nerve, and ulnar artery thrombosis.

**Interventions:**

The defect was closed with an ipsilateral ALT flap measuring 9 cm in length by 6 cm in width.

**Outcomes:**

After debridement was performed 3 times, the majority of the rectus femoris and lateral femoris were removed. Secondary closure by skin grafting was performed 2 weeks later. Three days after the procedure, necrotic tissues were noted on the edges of the wound. The diagnosis of ACS of the right was made. A second exploration was decided, and an extensive anterior compartment fasciotomy was performed. After 6 weeks of vacuum sealing drainage therapy, the defect was closed with a free latissimus dorsi musculocutaneous flap. At 4 months of follow-up, the right thigh wound had healed. At 6 months of follow-up, quadriceps muscle weakness remained. At 1 year of follow-up, the patient's mobility had been significantly improved, but diminished sensation remained on the lateral aspect of the thigh.

**Conclusion:**

ACS can occur after ALT flap harvesting in pediatric patients and should be recognized as early as possible to avoid devastating complications.

## 1 Introduction

Anterolateral thigh (ALT) flap has been proven to be a versatile flap for soft tissue reconstruction, thus gaining extensive popularity in recent years. Despite its extensive use, acute compartment syndrome (ACS) in pediatric patients has rarely been recorded in the literature, leading to devastating complications. We herein reported on a case of ACS occurring in a 6-year-old girl after ALT harvest with direct closure of the donor site.

## 2 Case presentation

**Figure 1:**
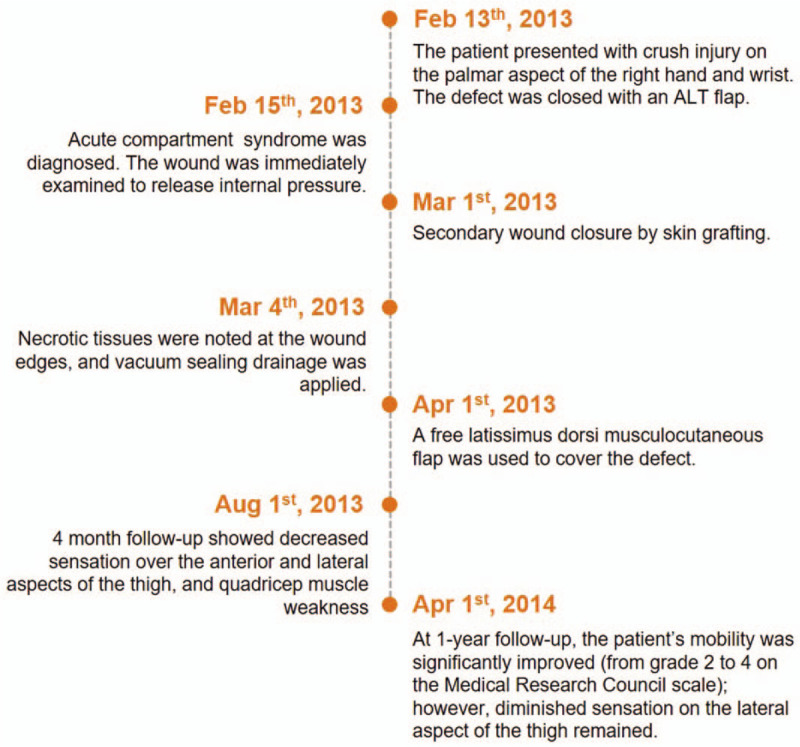
Patient timeline.

**Figure 2:**
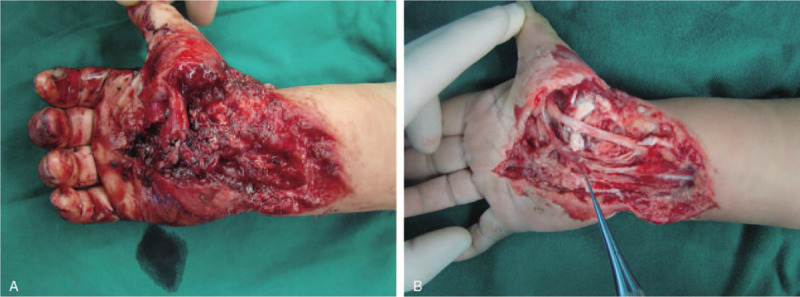
(A) Clinical appearance of the right hand and wrist (A). (B) Complete debridement was performed on the first day.

**Figure 3:**
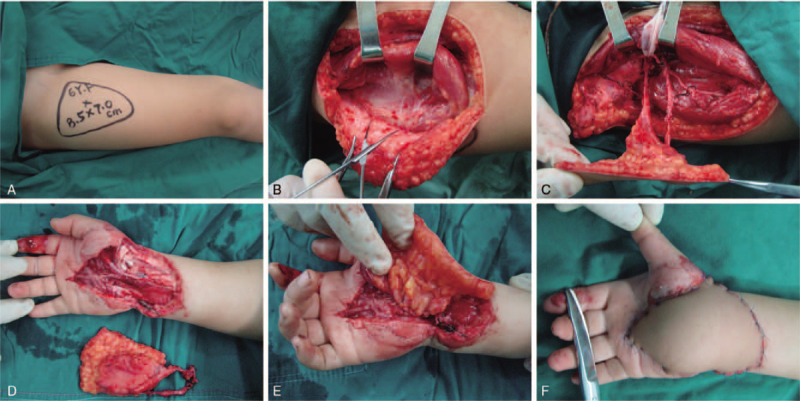
(A) Planning for the anterolateral thigh flap. (B-D) Dissection of the anterolateral thigh musculocutaneous flap. (E and F) The anterolateral thigh flap covered the soft tissue defect.

A 6-year-old Asian girl was admitted to the Second Hospital of Jilin University with crush injury on the palmar aspect of the right hand and wrist (Fig. 1). Emergency physical examination revealed stable vital signs, significantly swollen right hand with soft tissue defect, severely damaged thenar muscles, lightly crushed flexor tendons, crushed median nerve, and ulnar artery thrombosis (Fig. 2A). After debridement, the patient was noted to have tendon rupture of the flexor carpi radialis and the middle finger's flexor digitorum superficialis. There was a 5-cm defect of the ulnar artery and a 2-cm defect of the median nerve (Fig. 2B). The injury was associated with the rupture of the palmar side of the joint capsule. After radical debridement, an end-to-end repair of the median nerve was performed using an 8–0 polyamide suture. Then, muscles were readapted, and tendons were sutured. The size of the soft tissue defect in the proximal palm and wrist was 8 × 5 cm. The defect was closed with an ipsilateral ALT flap measuring 9 cm in length by 6 cm in width. The fascia lata was harvested with the flap to reconstruct the carpal joint capsule. Six-centimeter of the lateral femoral circumflex artery (LCA) was harvested along with the ALT flap to bridge the ulnar artery (Fig. 3). Routine management of the patient was performed.

**Figure 4:**
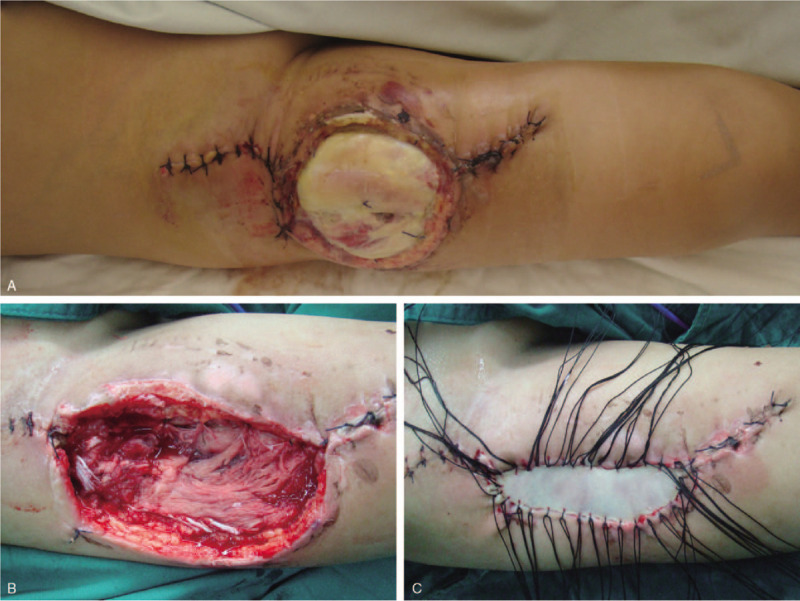
(A) Anterolateral thigh (ALT) donor site on postoperative day 14. (B) ALT donor site after debridement of dead muscle and marginal skin. (C) Skin graft on the donor site.

**Figure 5:**
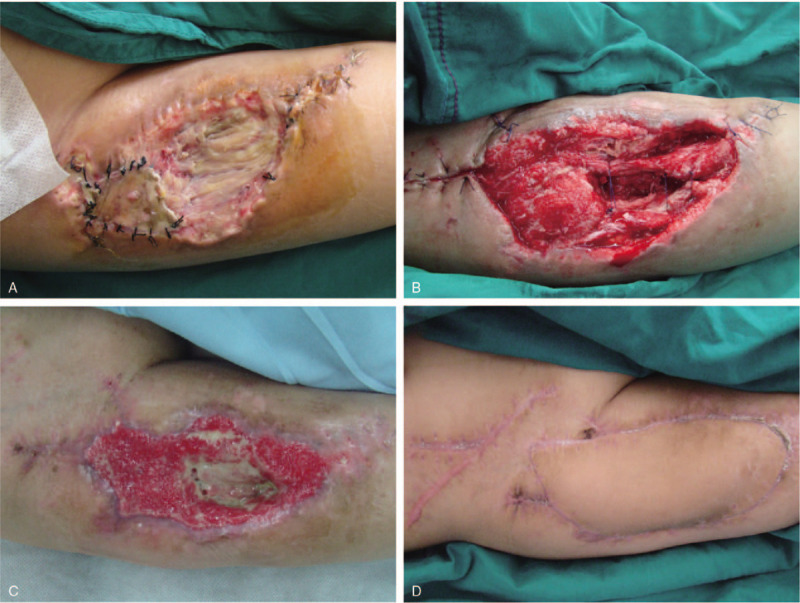
(A) Necrotic tissue on the wound edges. (B) A second exploration was performed to remove all necrotic tissues and surrounding fibrotic, scarred skin. (C) After 6 wk of vacuum sealing drainage therapy. (D) The final result of the fibula donor site after latissimus dorsi musculocutaneous flap repair.

The initial postoperative period was uneventful. On the first day after the operation, the patient was noted to have slight swelling of the right lower limb and redness of the thigh wound edges. The patient did not complain of pain or numbness, and the dorsalis pedis artery pulse was palpable. However, 40 hours after the operation, the patient complained of pain in the donor site and severe edema of the entire thigh. The patient was noted to have weakness of quadriceps muscles and necrosis and blistering of the thigh wound margins. Meanwhile, the patient complained of localized pain exacerbated by active and passive movement of the quadriceps. The diagnosis of ACS of the right thigh was supported and the wound was immediately explored to release the anterior compartment. All 4 muscles of the anterior compartment were considerably edematous and were released individually. Postoperatively, the patient was monitored in the intensive care unit. Two weeks later, an operative debridement revealed extensive partial necrosis of the rectus femoris and lateral femoris but no hematoma (Fig. 4A). A thorough debridement was performed, removing the majority of the rectus femoris and lateral femoris. Necrotic wound margins were also excised (Fig. 4B). Secondary closure by skin grafting was performed 2 weeks later (Fig. 4C). Three days after the procedure, necrotic tissues were noted on the wound edges (Fig. 5A). A second exploration was performed to remove all necrotic tissues and surrounding fibrotic, scarred skin (Fig. 5B). After 4 weeks of vacuum sealing drainage therapy, healthy granulation tissue was noted (Fig. 5C). The defect was closed with a free latissimus dorsi musculocutaneous flap measuring 9 cm in length by 5 cm in width. Over the next few days, the ALT flap survived completely (Fig. 5D). Sensory examination revealed sensory loss over the thenar area and the palm. Motor examination showed limited thumb opposition.

At 4 months follow-up, the right thigh wound had healed, but there was decreased sensation over the anterior and lateral aspects of the thigh. Motor examination showed that the patient had quadriceps muscle weakness (grade 2 of 5 on the Medical Research Council scale). At 1-year follow-up, the patient's mobility has been significantly improved (from grade 2 to 4 on the Medical Research Council scale); however, the diminished sensation on the lateral aspect of the thigh remained.

## 3 Discussion

We herein reported a case of ACS in a 6-year-old girl after ALT flap harvest with direct closure of the donor site. The ACS of the thigh is a severe condition resulting from increased pressures within any of the 3 thigh fascial compartments. Clinically, only the anterior fascial compartment is encountered when harvesting the ALT flap. The ALT flap is the most popularly used flaps for major soft tissue reconstruction because of the reliability and versatility of the soft tissues of the thigh, allowing the flap to fulfill most of the requirements of reconstruction of various body regions.^[1,2]^ Although it is widely used, ACS in pediatric patients has rarely been reported in the literature. Compartment syndrome develops when the tissue pressure inside the compartment exceeds the perfusion pressure of the arterial supply, leading to muscle and nerve ischemia. Addison et al^[2]^ reported 2 cases of sarcoma patients who developed ACS after ALT flap harvest and direct closure of the donor site. The diagnosis of ACS was delayed in both cases because of epidural analgesia, bed rest, and the lack of pain or particular signs. Two cases were noted to have blistering, tense, and necrotic marginal edges on postoperative days 3 to 5.^[2]^ Rare reports in the literature of an ACS developing at the ALT donor site was known, but it has been reported after direct closure of the fibular flap donor site.^[3]^

The diagnosis of ACS is difficult at any time point, even among expert surgeons. The symptoms are generally of localized pain exacerbated by passive stretching of involved muscles. Other signs and symptoms of ACS can include absent pulses, numbness, and paralysis. The diagnosis of ACS can be supported by the measurement of tissue stress, but are rarely used routinely.^[4]^ Tissue pressure in excess of 20 to 30 mm Hg requires careful follow-up. The emergent treatment is required when tissue pressure increases within 10 to 30 mm Hg of the diastolic compartment pressure.^[3]^ In this case, the wounds had been physically opened on the ward, and therefore, the compartment pressure was not measured.

Direct closure of the donor site under tension is likely to promote the development of ACS in the thigh. The factors include the flap width–to–thigh circumference ratio, skin laxity, and subcutaneous fat thickness.^[2]^ Most authors recommend a maximum width of 8 to 10 cm to close the donor site.^[5,6]^ Until now, the flap width in a pediatric patient has rarely been reported. In this case, the ALT flap harvested measured 9 cm in length by 6 cm in width. The skin of the pediatric patient was flabby, and no swelling or tension was noted after direct closure. No pain or numbness was reported during the early stage. However, 40 hours after the operation, the patient complained of pain in the donor site and severe edema of the whole thigh, with associated weakness of the quadriceps muscles and necrosis and blistering of the margins of the thigh wound. The patient developed an ACS that went unrecognized, leading to wide muscle necrosis and associated disability. This could have been contributed by issues of pediatric age, delayed pain, skin laxity, and flap width.

Because of variations in vascular anatomy, a muscle-related functional complication is common at donor sites following ALT flap harvesting.^[1]^ Necrosis of the rectus femoris was reported as a rare complication.^[1,7,8]^ The rectus femoris dominant pedicle comes from either the descending branch or the main trunk of the LCA. The ALT flap is harvested on the descending branch of the LCA. If the blood supply of the rectus femoris is disturbed and the other pedicle is insufficient, muscular atrophy or necrosis of the rectus femoris would develop. Maruccia et al^[8]^ suggested that the viability of the rectus femoris should be verified by temporarily interrupting the blood supply to the rectus femoris.

The predominant symptom is localized pain. However, pain may be mild or absent in a patient on bed rest who is being given analgesics. Moreover, sedation or major analgesics often “mask” the clinical symptoms of ACS, thus leading to an incorrect diagnosis. Some physicians believed that regional anesthesia could put the patient at higher risk for devastating complications.^[9]^ Preservation of the lateral femoral cutaneous nerve during ALT harvesting decreases the risk of ACS complications.

The challenge of diagnosing ACS in the pediatric population is an extreme challenge.^[10]^ The reasons for delayed diagnosis of ACS in the pediatric population are multi-factorial. The longer elapsed time between primary injury and peak compartment pressures have been reported in the pediatric setting.^[11]^ Besides, pediatric patients are too to cooperate. The combination of a related trauma, tight extremities, excessive pain sensations, pain on passive stretching of the muscle, sensory impairment, and an increasing analgesia requirement should give rise to a high suspicion. A high level of serum creatine phosphokinase (CPK) may be another supportive indicator for diagnosing ACS. Compartment pressure measurements might be needed. However, it is difficult to measure in an awake child and often require conscious sedation or anesthesia. Prompt fasciotomy and treatment of the underlying cause continue to be the recommended treatment. In this patient, direct closure of the donor site under excessive tension might be the cause of the ACS. The width of the skin paddle was only 6 cm, but the thigh was thin. Gharb et al.^[12]^ reported 5 cases of patients who covered with skin grafts with flap width ranging from 6 to 8 cm. Therefore, skin graft should be taken into account in this case. Muscle ischemia may be the other cause of the ACS, even if the donor site was closed without tension. Verifying the viability of the related muscles at the end of the surgery could avoid devastating complications.

Advances in microsurgical techniques have enabled replantation and soft tissue reconstruction with a high degree of success rate in children.^[13,14]^ Several authors have published a successful series of free tissue transfers in pediatric patients, confirming the safety and reliability of microsurgical reconstruction. However, the properties of pediatric reconstruction differ from those in adults. The perforators in younger children were significantly smaller than those in older children.^[12]^ Therefore, dissection of the perforators in younger children is more technically challenging. There are several options for soft tissue reconstruction in the upper extremity, including skin grafting, local flaps, regional flaps, and free flaps. In pediatric patients, skin graft and local flaps are well tolerated by patients and surgeons; hence, they remain as primary options. Free flaps are recommended only when these options could not meet the needs of the wound. Free flaps are also the preferred option in the reconstruction of the head, face, and lower limbs. In pediatric patients, soft tissue reconstruction is challenging because it affects the physical and esthetic appearance as well as the psychological and social impact to the patient. Hence, it was determined as the strategy that would best meet the needs of the wound and, eventually, the needs of the patients.

The patient's parents provided informed consent for the publication of this report.

## 4 Conclusion

ACS after anterolateral flap harvesting is possible, especially in pediatric patients, so skin graft closure might be used if primary closure would cause excessive tension. ACS should be recognized as early as possible in order to avoid devastating complications. Cautious postoperative care and monitoring are necessary, especially if compartment syndrome is suspected.

## Author contributions

**Conceptualization:** Wenrui Qu

**Investigation:** Heng Tian

**Methodology:** Junbo Pan

**Writing – original draft:** Wenrui Qu and Xuejie Wang

**Writing – review & editing:** Hongjuan Jin and Heng Tian
